# miR-27b-3p inhibits proliferation and potentially reverses multi-chemoresistance by targeting CBLB/GRB2 in breast cancer cells

**DOI:** 10.1038/s41419-017-0211-4

**Published:** 2018-02-07

**Authors:** Danni Chen, Wengong Si, Jiaying Shen, Chengyong Du, Weiyang Lou, Chang Bao, Huilin Zheng, Jie Pan, Guansheng Zhong, Liang Xu, Peifen Fu, Weimin Fan

**Affiliations:** 1 Program of innovative Cancer Therapeutics, Division of Hepatobiliary and Pancreatic Surgery, Department of Surgery, First Affiliated Hospital, College of Medicine, Zhejiang University, Hangzhou, 310000 China; 2 Key Laboratory of Organ Transplantation, Zhejiang, 310000 China; 3 Key Laboratory of Combined Multi-organ Transplantation, Ministry of Public Health, Hangzhou, 310000 China; 4 Breast Center, First Affiliated Hospital Zhejiang University, College of Medicine, Hangzhou, 310000 China; 5 Clinical Research Center, First Affiliated Hospital of Zhejiang University College of Medicine, Hangzhou, 310000 China; 6 Department of Pathology and Laboratory Medicine, Medical University of South Carolina, Charleston, SC 29425 USA

## Abstract

Drug resistance remains a major problem in the treatment of conventional chemotherapeutic agents in breast cancers. Owing to heterogeneity and complexity of chemoresistance mechanisms, most efforts that focus on a single pathway were unsuccessful, and exploring novel personalized therapeutics becomes urgent. By a system approach, we identified that microRNA-27b-3p (miR-27b), a miRNA deleted in breast cancer tissues and cell lines, has a master role in sensitizing breast cancer cells to a broad spectrum of anticancer drugs *in vitro* and *in vivo*. Mechanistic analysis indicated that miR-27b enhanced responses to PTX by directly targeting CBLB and GRB2 to inactivate both PI3K/Akt and MAPK/Erk signaling pathways. Further, miR-27b was identified as a promising molecular biomarker in chemoresistance, clinicopathological features, and prognosis for breast cancer patients. In conclusion, we propose that combinational use of miR-27b and chemotherapeutic agents might be a promising therapeutic strategy to increase long-term drug responses in breast cancers.

## Introduction

Breast cancer is one of the most commonly diagnosed cancers in female malignancies; breast cancer alone is expected to account for 29% in all new cancer diagnoses in 2016^1^. A significant increase in breast cancer incidence was reflected since the 1980s because of changes in female reproductive patterns and the increased detection of asymptomatic disease during the rapid uptake of mammography screening^2^. In 2016, breast cancer accounted for 14% of all cancer deaths, secondary to lung and bronchus, and the disease burden is becoming increasingly severe because of more exposure to young women^1^. Chemotherapy is one of the most important therapeutic strategies for breast cancer, especially in developing countries; however, a successful long-term of chemotherapy is often prevented by development of drug resistance and adverse effects of antitumor drugs^3^. Acquired chemoresistance is more common in clinical practice, which means tumors are initially sensitive to chemotherapeutic agents while gradually acquire chemotherapeutic resistance during treatment^4^. What is worse, once tumors get resistance to one specific anticancer drug, they always exhibit resistance to a broad range of other chemotherapeutic drugs, chemically and functionally unrelated, called multi-chemoresistance^4,5^. Thus, it is desirable that a novel therapeutic strategy should be developed to rescue multi-chemoresistance and enhance the effectiveness of anticancer drugs.

Recent studies demonstrated that drug resistance is regulated not only by genetic and epigenetic changes, but also by microRNAs (miRNAs)^6^. MiRNAs are a group of small, non-coding RNAs, which are single-stranded and consist of 19–25 nucleotides (~22 nt). They regulate genes expression through modulation of post-transcriptional activity, by binding to the 3′-untranslated region (UTR) of multiple target mRNAs in a sequence-specific manner^7,8^. Numerous miRNAs have been shown to regulate diverse biological process, such as proliferation, cell cycle control, apoptosis, migration, and metabolism^9–11^. Furthermore, miRNAs have been shown to be dysregulated in various tumors, resulting in aberrant expression of target proteins, indicated that they could function as tumor suppressors or oncogenes^12^. Finally, recent studies showed that up- or downregulation of miRNAs influenced variations in chemosensitivity of cancer cells via diverse cellular processes^13^. Most miRNAs have been showed to modulate chemotherapeutic resistance by regulating survival pathways and/or apoptosis pathways^13,14^. For example, miR-199a-3p enhanced cell sensitivity to doxorubicin by antagonizing mTOR and c-Met^15^. miR-181a and miR-630 enhanced cisplatin-induced cancer cell death in non-small cell lung cancer (NSCLC) cells^16^. In contrast, some miRNAs may exhibit oncogenic activity and induce drug resistance. In breast cancer, miR-21 was found overexpressed, resulting downregulation of tumor suppressor protein PDCD4. The alteration lead to overexpression of inhibitors of apoptosis proteins and multidrug-resistant protein 1, resulting reduced apoptosis and chemotherapeutic resistance^17^. In liver cancers, miR-221/222 induced TNF-related apoptosis-inducing ligand resistance by targeting tumor suppressors PTEN and TIMP3^18^. These studies demonstrated that the functions of miRNAs might be complicated and even contrast in different tumors. Importantly, two miRNAs-based drugs, miR-34a and miR-122, have entered clinical trials in patients with liver cancers^19,20^. The evidence strongly indicated that miRNA-based combinational therapy has potential to be developed into a novel treatment to achieve long-term drug responses in clinical practice. Apparently, how to identify efficient miRNAs that play an important role in multiple essential drug resistance pathways, to achieve potent drug responses, becomes an urgent need. Recently, a study have identified that miR-27b-3p (also known as miR-27b) functioned as a tumor suppressor to inhibit breast cancer stem cell generation by inactivating ENPP1, to attenuate chemoresistance and tumor seeding ability^21^. Moreover, another study confirmed miR-27b as a master miRNA to improve the sensitivity to a broad spectrum of anticancer drugs by activating p53-dependent apoptosis and reducing CYP1B1-mediated drug detoxication in a defined subgroup of liver and kidney cancer patients^22^. However, in breast cancer, miR-27b was reported to have a cancer-promoting role and associated with poor prognosis in triple-negative breast cancer patients^23^. Conversely, downregulation of miR-27b was showed to enhance tamoxifen resistance by increasing NR5A2 and CREB1 expression^24^. These findings suggested that functions of miR-27b might be diverse and depend on specific cancer type, and the mechanism of regulation chemosensitivity or -resistance was unclear.

In current study, we found that miR-27b genetically decreased both in breast cancer tissues and cell lines, and its downregulation was associated with chemoresistance, malignancy, and poor prognosis. In our experiments, miR-27b significantly inhibited cell proliferation and colony formation, promoted apoptosis and generated synergistic effect with a broad range of anticancer drugs *in vitro*. Meanwhile, the synergistic effect with paclitaxel (PTX) was also efficient *in vivo*. Further, we demonstrated for the first time that miR-27b reversed PTX-resistance by directly downregulating its target genes, CBLB and GRB2, thereby inactivating MAPK/Erk signaling pathways and PI3K/Akt signaling pathways, which are classic pathways involved in multi-chemoresistance. In conclusion, our results confirmed that miR-27b served potentially as effective biomarkers for prediction of therapeutic effect and prognosis, importantly, as effective targets for combinational therapy with chemotherapeutic agents as novel treatment strategies against breast cancers.

## Results

### miR-27b expression is genetically decreased in breast cancer and reversely associated with chemoresistance, malignancy, and poor prognosis

**Fig. 1:**
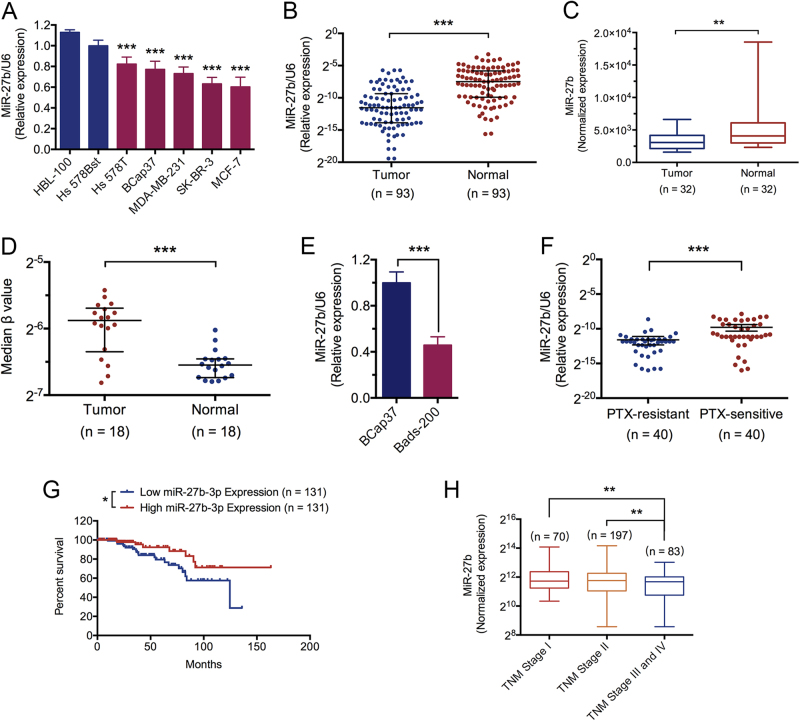
Downregulation of miR-27b correlates with chemoresistance, malignancy, and poor prognosis in breast cancer. **a** The miR-27b expression in five breast cancer cell lines (Hs578T, BCap37, MDA-MB-231, SK-BR-3, MCF-7) were compared with that in two breast cell lines (HBL-100, Hs578Bst). **b**, **c** The miR-27b expression in tumor tissues was compared with that in adjacent normal tissues in luminal A-breast cancer patients from clinical and TCGA database. **d** The promoter methylation level of the miR-27b gene in breast cancer patients from TCGA database. **e**, **f** The association of miR-27b expression and PTX-resistance were measured in breast cancer cells and tumor tissues from clinical breast cancer patients. **g** Kaplan–Meier survival curves of early-stage (TNM stages I and II) luminal A-breast cancer patients according to miR-27b expression level. **h** The correlation analysis between miR-27b levels and the TNM stage in luminal A-breast cancer patients

To determine the expression of miR-27b in breast cancer cells, we analyzed miR-27b expression by qRT-PCR and found miR-27b was markedly downregulated in breast cancer cell lines (Fig. 1a). Then we compared the expression of miR-27b in breast cancer tissues and their matched adjacent normal breast tissues in luminal-type breast cancer patients (*n* = 93) and found that tumor tissue specimens exhibited generally lower miR-27b levels (Fig. 1b). Further, similar results were observed in luminal A breast cancer patients (*n* = 32) from The Cancer Genome Atlas (TCGA), which has set up vigorous criteria for human cancer sample collection and information processing^25^. (Fig. 1c). Importantly, by assessing epigenetic alteration at miR-27b promoter locus in samples from TCGA (*n* = 18), we found that the promoter locus status of miR-27b expressed significant hypermethylation in tumor tissues (Fig. 1d). Moreover, we analyzed miR-27b expression in BCap37 and the established PTX-resistant subline, Bads-200^26^, and found that miR-27b expression was significantly lower in Bads-200 cells (Fig. 1e). Further, the reverse association of miR-27b with chemoresistance was confirmed in tumor tissues from PTX-resistant group and PTX-sensitive group (*n* = 40, respectively) (Fig. 1f). In addition, we found that patients with low miR-27b level had significantly poorer survival than that with high miR-27b expression (*n* = 262, luminal A, Tumor Node Metastasis (TNM) stage I and II) (Fig. 1g), and downregulation of miR-27b expression was associated with advanced stage (*n* = 350, luminal A) (Fig. 1h).

### miR-27b suppresses cell proliferation, colony formation, and promotes chemosensitivity and PTX-induced apoptosis

**Fig. 2:**
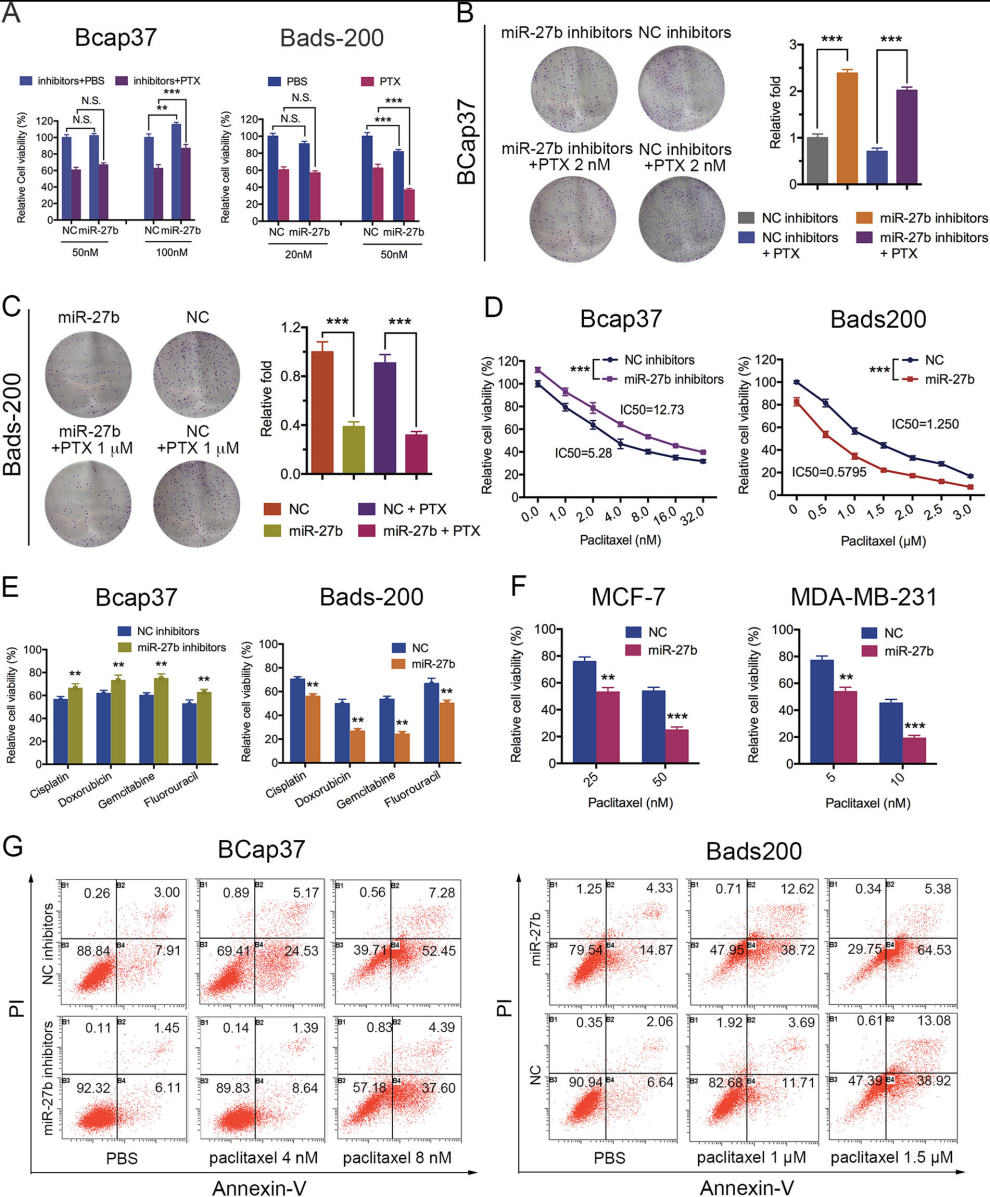
miR-27b suppresses cell proliferation, colony formation, and promotes chemosensitivity and PTX-induced apoptosis. **a**, **b**, **c** BCap37 cells and Bads-200 cells were transfected with miR-27b mimics (miR-27b) or miR-27b inhibitors, respectively. Cell viability assays, colony formation assays were measured. **d**, **e** The sensitivity of BCap37 and Bads-200 cells to multiple anticancer drugs were measured. (BCap37 cells: cisplatin 1 μΜ, doxorubicin 100 nM, gemcitabicin 500 nM, fluorouracil 1.2 μΜ. Bads-200 cells: cisplatin 1 μΜ, doxorubicin 10 μΜ, gemcitabicin 1 μΜ, fluorouracil 2 μΜ). **f** The sensitivity of MCF-7 and MDA-MB-231 cells to PTX were measured. **g** The apoptotic rate of BCap37 and Bads-200 cells with specific treatments were measured by flow cytometry analysis

Bads-200 cells were lipotransfected of miR-27b mimics (miR-27b), whereas BCap37 cells were lipotransfected of miR-27b inhibitors, respectively, (Supplementary Figure 1 A, 1B). The cell viability assays showed that the treatment of miR-27b had a notable reduced cell viability in Bads-200 cells, whereas miR-27b inhibitors had a significant increased cell viability in BCap37 cells. Moreover, combinational treatment of miR-27b and PTX markedly inhibited cell viability in Bads-200 cells, whereas miR-27b inhibitors decreased PTX sensitivity in BCap37 cells (Fig. 2a). The colony formation assay further confirmed that miR-27b significantly reduced clonogenicity of Bads-200 cells, whereas miR-27b inhibitors notably increased clonogenicity of BCap37 cells(Fig. 2b,c). The following MTT analysis showed that miR-27b can significantly downregulate the IC50 of Bads-200 cells to PTX, whereas miR-27b inhibitors notably upregulate the IC50 of BCap37 cells (Fig. 2d). Markedly, we found same observation in combination of various anticancer drugs, including Cisplatin, Doxorubicin, Gemcitabine, Fluorouracil (Fig. 2e). In addition, the drug-sensitizing effect could be extended to MCF-7 and MDA-MB-231 cells (Fig. 2f). Moreover, miR-27b were found to promote cell apoptosis with or without PTX, whereas miR-27b inhibitors decreased cell apoptosis (Fig. 2g).

### miR-27b suppresses tumorigenesis and promotes therapeutic effect with PTX of breast cancer *in vivo*

**Fig. 3:**
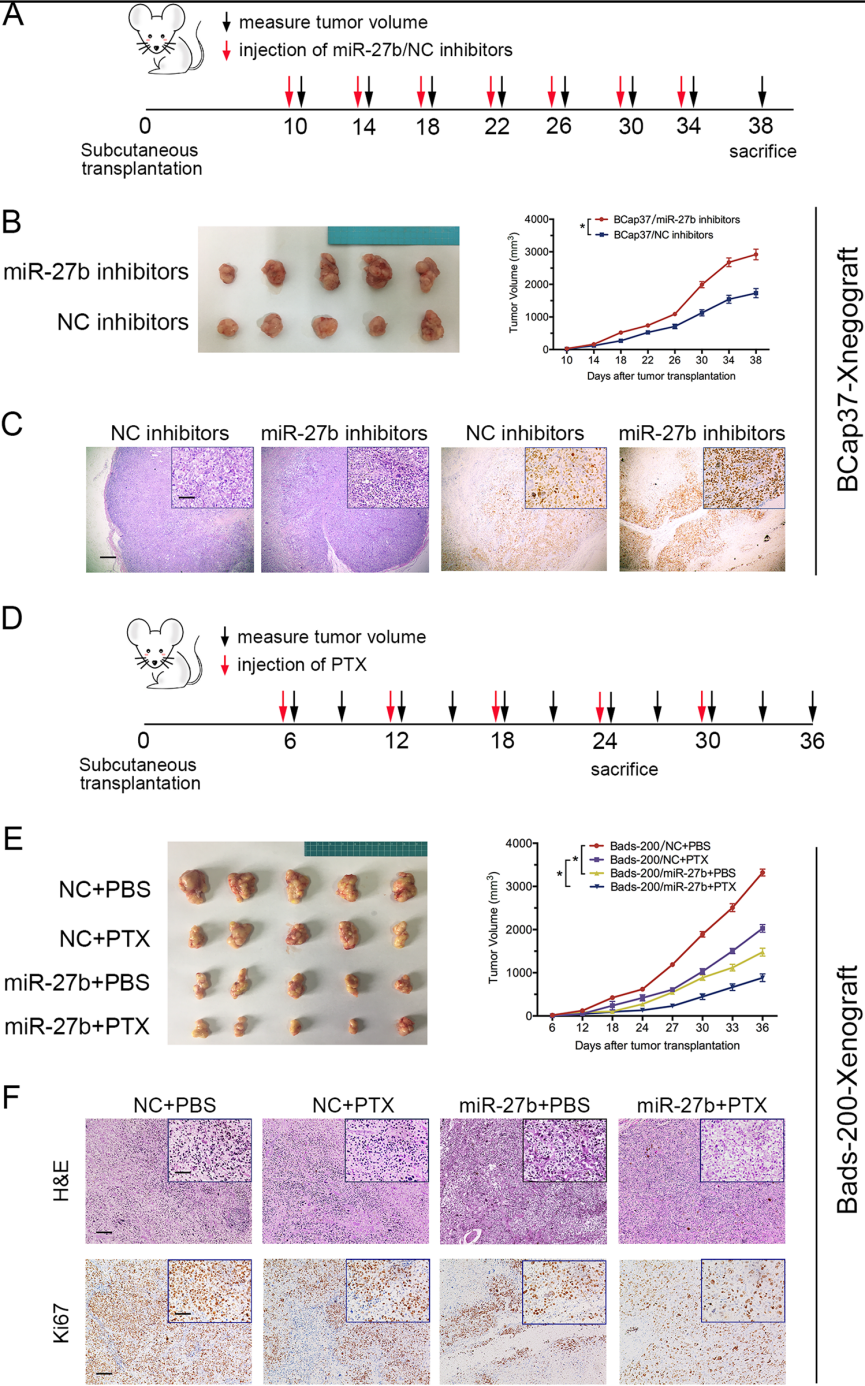
miR-27b suppresses the tumorigenesis and promotes therapeutic effect with PTX of breast cancer *in vivo*. **a**, **b** The xenograft were built by BCap37 cells transfected with miR-27b inhibitors with subsequent treatment of miR-27b inhibitors. The tumor volumes were measured and quantified. **d**, **e** The xenograft were built by Bads-200 cells stable expressing miR-27b with following treatment of PTX. **c**, **f** Representative images of tumor samples that were stained with hematoxylin and eosin (H&E) and Ki67 by IHC. Scale bars: (main) 100 μm; (insets) 25 μm

BCap37 cells were transfected with miR-27b inhibitors(Supplementary Figure 1B) and subcutaneous transplanted into nude mice with subsequent treatment of miR-27b inhibitors (Fig. 3a). In BCap37-Xenograft, significant induced tumorigenesis was found in miR-27b knockdowned group (Fig. 3b). Hematoxylin and eosin (H&E) and Ki67 staining further showed that cell proliferation was increased, whereas apoptosis was decreased by miR-27b inhibitors (Fig. 3c). On the other hand, Bads-200 cells were transfected with miR-27b overexpressing vector to establish miR-27b expressing stable cell line (Supplementary Figure 1E). All mice were randomly divided into four groups treated with PTX or posphate-buffered saline (PBS) every 6 days (10 mg/kg) (Fig. 3d). The mean volume of tumors of the miR-27b expressing groups was obviously smaller than that in the negative control (NC) group. Further, tumors in combination of miR-27b and PTX group showed greater suppression than that in PTX group (Fig. 3e). In addition, H&E staining implied the cell proliferation was inhibited in miR-27b stable expressing group, and Ki67 staining showed that combinational use of miR-27b and PTX significantly increased apoptosis (Fig. 3f).

### CBLB and GRB2, as candicate target genes of miR-27b, are inversely correlated with chemosensitivity and miR-27b

**Fig. 4:**
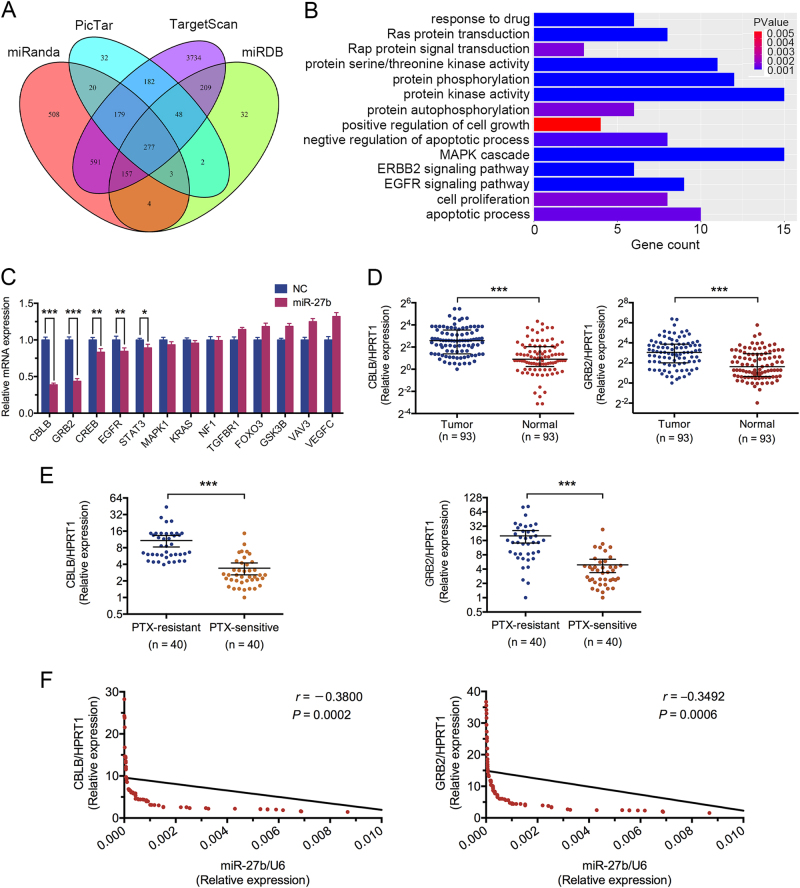
The expression of CBLB and GRB2 are inversely correlated with chemosensitivity and miR-27b expression. **a**, **b** Candidate target genes of miR-27b were screened and functional pathways were predicted by GO analysis. **c** The mRNA expressions of 13 candidate target were analyzed in Bads-200 cells. **d** The mRNA expression levels of CBLB and GRB2 were analyzed in breast cancer patients (*n* = 93). **e** The expressions of CBLB and GRB2 in PTX-resistance and PTX-sensitive groups (*n* = 40, respectively). **f** The correlations of miR-27b expression and CBLB/GRB2 expressions were analyzed in tumor tissues, respectively (*n* = 93)

To identify targets of miR-27b that involved in miR-27b-mediated regulation of chemoresistance in breast cancer, we searched four online miRNA target bioinformatics prediction databases (miRanda, PicTar, TargetScan, and miRDB). Initially, 277 potential genes of miR-27b were predicted in all four databases (Fig. 4a). Functional analysis by Kyoto Encyclopedia of Genes and Genomes (KEGG) and Gene oncology (GO) (https://david.ncifcrf.gov/) showed most of target genes had relation with cancer-related pathways and drug response pathways (Fig. 4b, Supplementary Figure 2 A). As shown in Fig. 4c, the mRNA expressions of five genes (CBLB, GRB2, CREB, EGFR, STAT3) among 13 potential candidate genes were found downregulated by miR-27b. Next, we compared mRNA levels of these genes in tumor tissues and their matched adjacent normal tissues in breast cancer patients (*n* = 93) and found CBLB and GRB2 expression were significantly higher in tumor tissues (Fig. 4d). Similar results were observed in patient tissues from TCGA database (Supplementary Figure 2E, 2 F). Although, CREB/EGFR/STAT3 were not observed such results in breast cancer patients (Supplementary Figure 2B, 2 C, 2D). Intriguingly, CBLB and GRB2 expression were significantly lower in PTX-sensitive group than PTX-resistant group, which indicated a reverse relation between CBLB/GRB2 expression and PTX sensitivity (*n* = 40, respectively) (Fig. 4e). Moreover, a significantly negative correlation between miR-27b and CBLB/GRB2 expression were found in tumor tissues of breast cancer patients (*n* = 93) (Fig. 4f).

### CBLB and GRB2 are direct targets of miR-27b

**Fig. 5:**
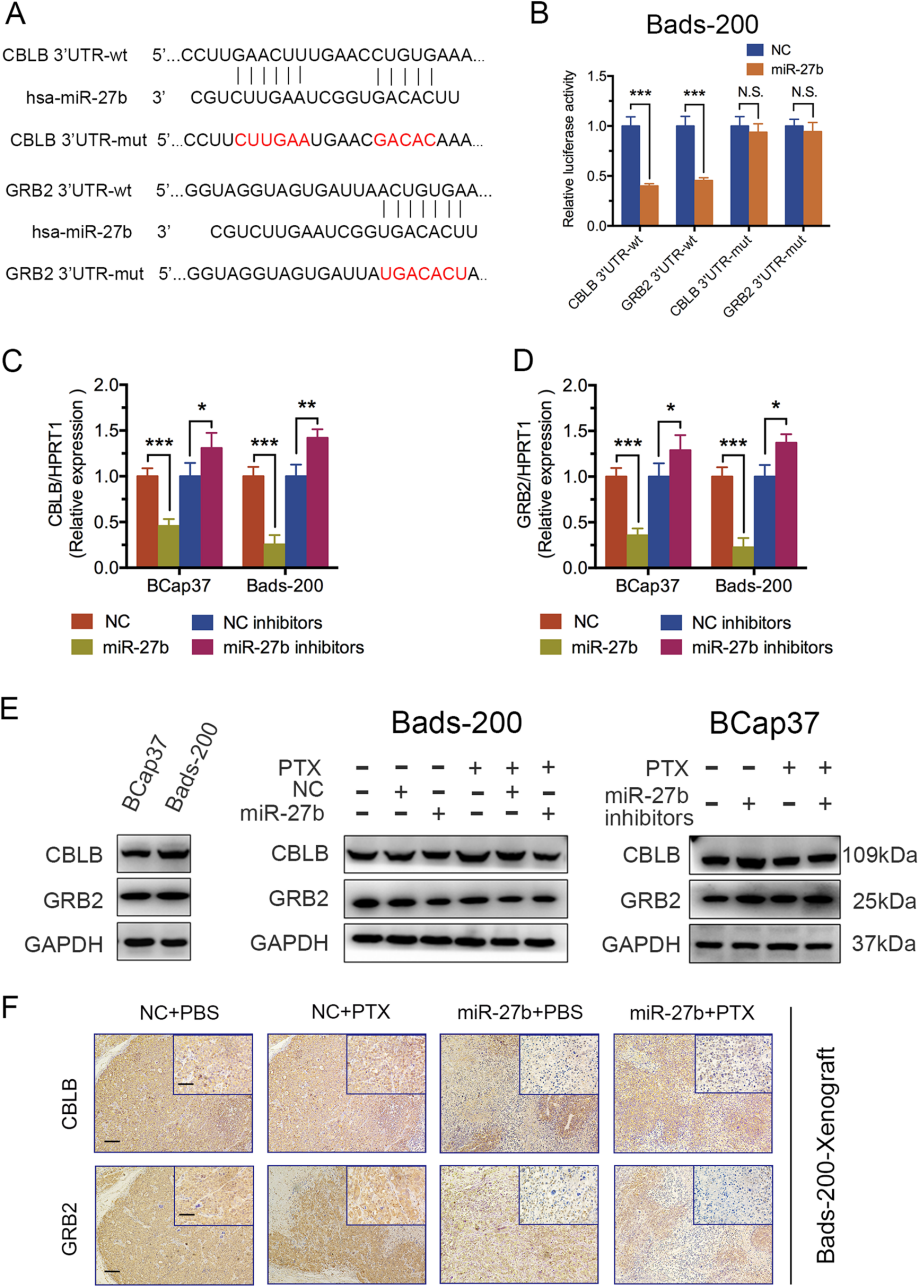
CBLB and GRB2 are direct targets of miR-27b. **a** Predicted sequences binding regions between wild-type (wt) or mutant (mut) 3′-UTRs of CBLB/GRB2 and miR-27b. **b** The luciferase activities were measured in Bads-200 cells co-transfected with miR-27b and specific luciferase vectors. **c**, **d**, **e** The mRNA and protein levels of CBLB/GRB2 in BCap37 and Bads-200 cells were analyzed. **f** Representative images of tumor samples in Bads-200-xenograft that were stained with CBLB and GRB2 by IHC. Scale bars: (main) 100 μm; (insets) 25 μm

The dual-luciferase assay was performed to figure out whether CBLB and GRB2 were direct targets of miR-27b. The binding sites of miR-27b on 3′-UTRs of CBLB and GRB2 were shown in Fig. 5a. The luciferase reporter assay showed miR-27b significantly decreased the luciferase activities of the wild-type 3′-UTR reporters of CBLB and GRB2 in Bads-200 cells, whereas no obvious reduction was observed in the luciferase activities of the mutant-type 3′-UTR reporters of CBLB/GRB2 (Fig. 5b). The results strongly indicated that CBLB and GRB2 are direct targets of miR-27b. Moreover, the mRNA expressions of CBLB/GRB2 were found significantly decreased by miR-27b, whereas increased by miR-27b inhibitors, both in BCap37 and Bads-200 cells (Fig. 5c,d). As shown in Fig. 5e, the protein expression levels of CBLB and GRB2 were compared between parent and resistant cells (BCap37 and Bads-200 cells). The results showed that CBLB and GRB2 expression were both lower in BCap37 cells, which further indicated the reverse association between CBLB/GRB2 expression and PTX sensitivity. Intriguingly, miR-27b significantly attenuated protein levels of CBLB and GRB2 in Bads-200 cells, whereas miR-27b inhibitors observably enhanced the protein expression level of CBLB and GRB2 in BCap37 cells, with or without PXT (Fig. 5e). In addition, we also confirmed the association in the tumors in Bads-200-Xenograft and BCap37-Xenograft by immunohistochemistry staining (Fig. 5f, Supplementary Figure 2 G). Above results definitely indicated miR-27b negatively regulated CBLB and GRB2 by direct binding in breast cancer cells.

### miR-27b inhibits proliferation and resistance to PTX of breast cancer cell by repressing CBLB and GRB2

**Fig. 6:**
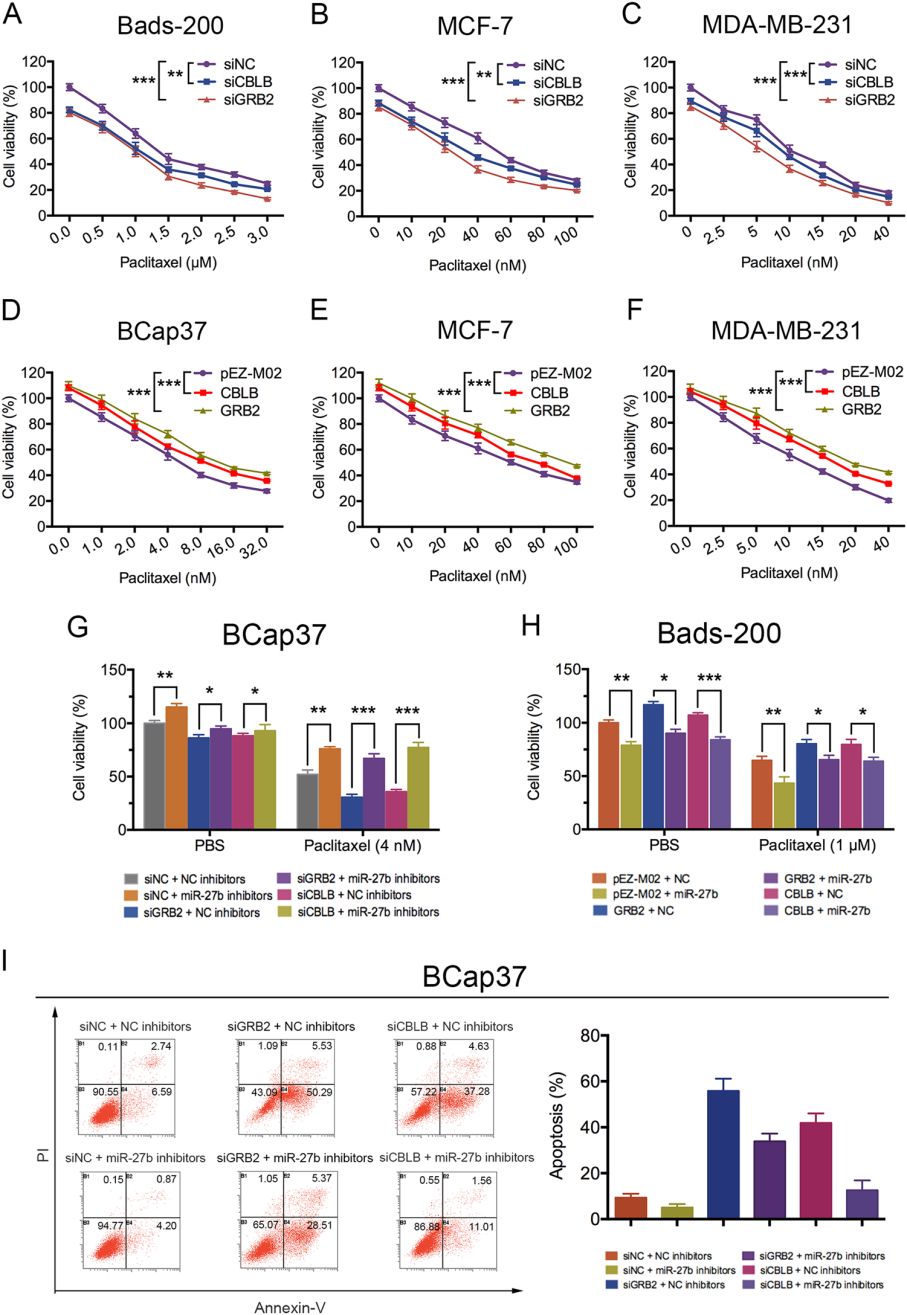
miR-27b inhibits cell proliferation and resistance to PTX of breast cancer cells by repressing CBLB and GRB2. **a**, **b**, **c** Depletion of CBLB and GRB2 reversed cell resistance to PTX in various breast cancer cells. **d**, **e**, **f** Overexpression of CBLB and GRB2 induced cell resistance to PTX in various breast cancer cells. **g**, **h** Cell viability assays were measured both in BCap37 and Bads-200 cells according to specific treatments. **i** The apoptosis rates of BCap37 cells with specific treatments were measured by flow cytometry analysis

The MTT assays showed downregulation of CBLB or GRB2 expression significantly inhibited cell proliferation, and increased cell sensitivity to PTX in breast cancer cells (Fig. 6a,b,c). In addition, overexpression of CBLB or GRB2 moderately induced cell proliferation, and decreased cell sensitivity to PTX (Fig. 6d,e,f). Furthermore, BCap37 cells were co-transfected with miR-27b inhibitors along with CBLB/GRB2 siRNAs. Cell viability assay indicated depletion of CBLB and GRB2 obviously reversed the PTX-resistant effects of miR-27b inhibitors in BCap37 cells (Fig. 6g). In addition, cell viability assay showed PTX-inhibited cell viability was substantially increased by miR-27b, whereas CBLB and GRB2 overexpression significantly reversed the PTX-sensitizing effects of miR-27b in Bads-200 cells (Fig. 6h). Moreover, flow cytometry analysis showed that reduction of PTX-induced apoptosis by miR-27b inhibitors was totally reversed by CBLB/GRB2 siRNAs in BCap37 cells (Fig. 6i). All these results suggested that miR-27b attenuated breast cancer cell resistance to PTX by repressing CBLB and GRB2.

### miR-27b suppresses activities of PI3K/AKT and MAPK/ERK signaling pathways through downregulation of CBLB and GRB2

**Fig. 7:**
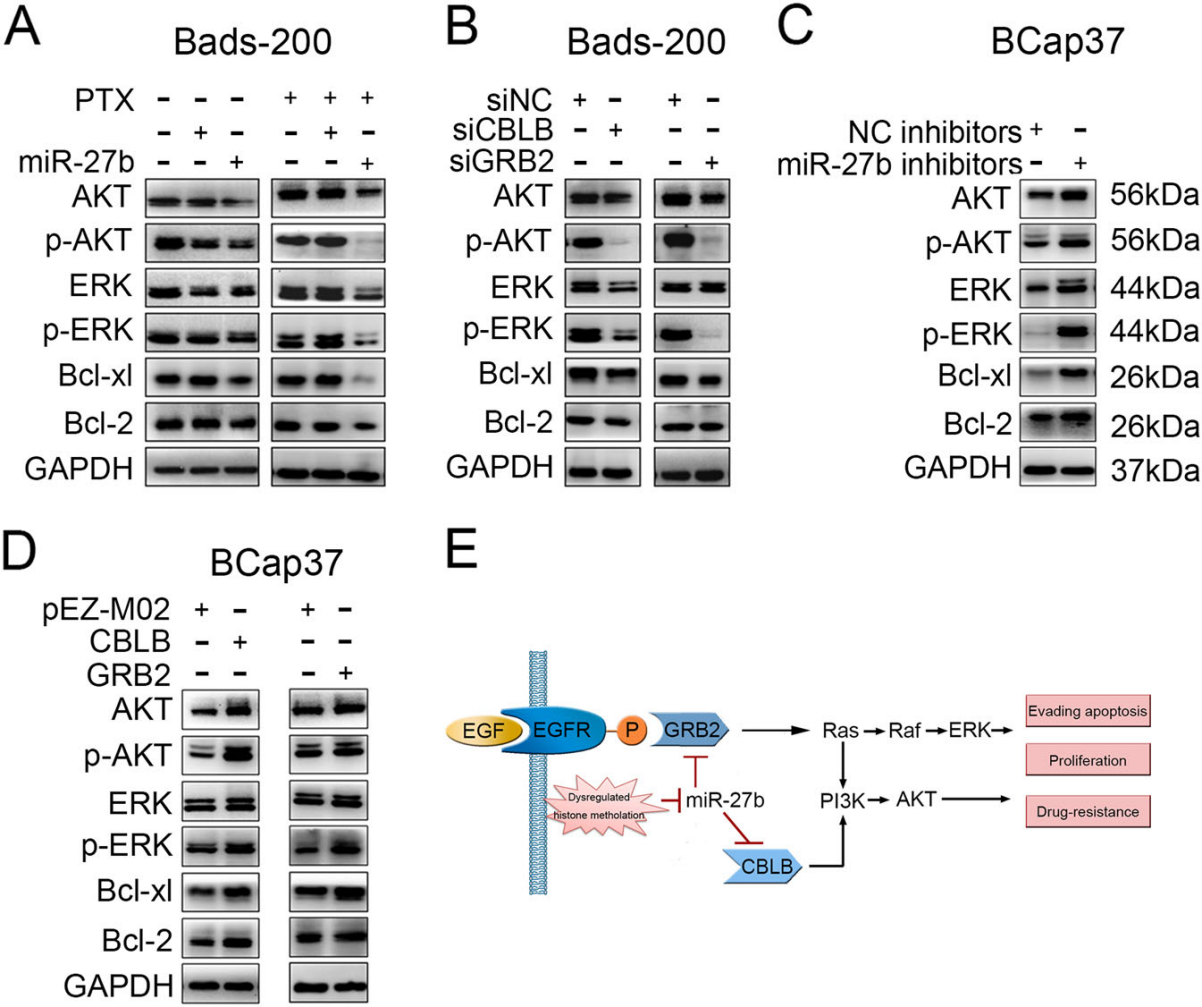
Expression of CBLB and GRB2 downstream proteins, anti-apoptosis proteins were regulated by miR-27b. **a**–**d** The protein levels of AKT, p-AKT, ERK, p-ERK, Bcl-2, and Bcl-xl in BCap37 and Bads-200 cells were measured by western blotting according to specific treatments. **e** Schematic model of miR-27b as a role in regulating functions in breast cancer cells

To further investigate the mechanisms underlying the effects of miR-27b, the expression of AKT, phosphorylated AKT (p-AKT), ERK, phosphorylation of ERK (p-ERK), downstream proteins of CBLB and GRB2 pathways, and apoptosis-related proteins were determined by western blotting. In Bads-200 cells, miR-27b was found significantly downregulate downstream proteins of CBLB and GRB2 pathways, and anti-apoptosis proteins, Bcl-2 and Bcl-xl. Interestingly, these proteins level were expressed at significantly lower levels in cells with combinational treatment of miR-27b and PTX (Fig. 7a). Similarly results were observed in Bads-200 cells transfected with CBLB or GRB2 small interfering RNAs (siRNAs) (Fig. 7b). In contrast, miR-27b inhibitors could increase the AKT, p-AKT, ERK, and p-ERK expression in BCap37 cells. The anti-apoptosis proteins expressed at higher level by miR-27b inhibitors and CBLB/GRB2 overexpression plasmids (Fig. 7c,d). In summary, miR-27b suppresses CBLB and GRB2, thereby inactivating of PI3K/Akt and MAPK/Erk signaling pathways and anti-apoptosis proteins.

## Discussion

Chemotherapy is an important therapeutic strategy for breast cancer, especially in developing countries. However, chemotherapy fails to eliminate all tumor cells because of acquisition of anticancer resistance^27^. In recent years, accumulating studies have suggested miRNAs play important roles in the regulation of drug sensitivity via miRNA-based mechanisms^28–30^. Previous studies showed the expressions of miR-200b, miR-194, and miR-212 were significantly downregulated, whereas the expression of miR-192, miR-424, and miR-98 were significantly upregulation in docetaxel-resistant NSCLC cells^31^. It suggested that different miRNAs were involved in docetaxel-resistance. Another study showed overexpression of miR-320 and miR-204 was reported to sensitize cholangiocarcinoma cells to 5-Fluorouracil by suppressing Mcl-1, a member of Bcl-2 family^32^. In addition, miR-181a and miR-630 regulated cisplatin-induced cancer cell death in NSCLC cells^33^. Increased expression of miR-21 was reported to lead to a significant reduction of apoptotic index in both control cells and cells treated with gemcitabine^34^. Above researches have confirmed miRNA as a promising target in drug resistance, whereas considering the property of miRs might be cell and tissue dependent, and its potential role in chemoresistance should be contextualized with respect to tumor type and treatment. In this study, we found that miR-27b expression markedly decreased in luminal-type breast cancer tissues and various breast cancer cell lines. Intriguingly, downregulation of miR-27b significantly related with PTX-resistance both in breast cancer cell lines and tissues. These observations suggested miR-27b might be a disadvantage for tumors and chemotherapy-induced resistance. Recent studies have reported variations in miRNA regions or dysregulation of miRNA-processing pathways may affect the expression levels of mature miRNAs, and functions in drug sensitivity^35^. Therefore, the pharmacogenetic analysis of miRNAs may present an innovative field of research for predicting treatment response or chemoresistance^36^. In current study, the significance of miR-27b was further highlighted by the finding that mir-27b gene locus is hypermethylation in human breast cancers based the data derived from TCGA database, which might explain the lower expression of miR-27b in malignant and chemoresistant tissues and cells, the great synergistic effect of miR-27b in PTX-resistant cells *in vivo*. Importantly, the low miR-27b may confer an inducer on cancer cells and restoring miR-27b expression may selectively kill cancer cells in combination with anticancer drugs. Notably, in our study, downregulation of miR-27b was the first time indicated to be association with advanced tumor stage and poor survival, which suggesting miR-27b may serve as a molecular prognostic marker for breast cancer aggressiveness, and the expression profiles of miR-27b could provide information about resistance of individual tumors to different treatments, before starting therapy.

Recently, miRNA therapeutic appears as a novel field in which miRNA activity and function are the major targets of the intervention. Owing to the heterogeneity and complexity in cancer cells and different actions of various anticancer drugs, multiple mechanisms are involved in chemoresistance in clinic. Most of studies focus on single miR-based pathway or single anticancer drug, which might be unable to produce sustained drug response and be a deficiency in applying miR-based treatment in clinical practice^37^. In our study, we confirmed that miR-27b remarkably increased drug responses breast cancers cells, especially PTX-resistant cells (Bads-200), to various anticancer drugs (PTX, cisplatin, doxorubicin, gemcitabine, and flurouracil). *In vivo*, Bads-200-xenograft, which coincided with chemoresistance in clinic, similar effects were also observed, further confirming a promising potent of miR-27b as a novel use to overcome multiple antidrug resistances. In conclusion, our results provide a network-based perspective to understand the role of miR-27b as a master regulator in reversing multiple chemoresistance.

Remarkably, our study indicated CBLB and GRB2 were direct targets of miR-27b, and played important roles in miR-27b-mediated PTX sensitivity in breast cancers. CBLB, an upstream factor in PI3K/Akt signaling pathways, was demonstrated as an important factor participate in ubiquitination to regulate multiple cell processes. It reported that CBLB regulated the sensitivity of cetuximab through ubiquitin-proteasome system in human gastric cancer cells^38^. Another study also reported that downregulation of CBLB sensitized gastric cancer to rapamycin through PI3K/Akt pathway^39^. In breast cancers, CBLB was reported to regulate p-glycoprotein transporter function^40^. Although the mechanisms underlying reversion of multiple chemoresistance in breast cancers were not clarified. In current study, we uncovered the downregulation of CBLB in miR-27b-mediated drug sensitivity, thereby suppressing PI3K/Akt pathway activities. Various studies have showed the PI3K/Akt signaling pathways participated in multiple cellular processes, such as proliferation, migration, apoptosis. Downregulation of PI3K/Akt pathway activities was also reported to enhance the antitumor effect of PTX against chemoresistant ovarian cancer cells^41^. These findings are consistent with our study and further sustain the functions of CBLB underlying miR-27b-mediated tumor chemosensitivity in breast cancers. Moreover, GRB2, an important upstream factor in MAPK/Erk signaling pathways, was reported to have implicated in resistance of ovarian cancer to cisplatin by activating MAPK/Erk signaling pathways^42^. The MAPK/Erk signaling pathways were reported to be involves in multidrug resistance by rendering cells less susceptive to apoptosis^43^. However, in breast cancer, the functions of GRB2 underlying miR-27b-mediated drug sensitivity were not clear. In this study, we first time confirmed GRB2, as direct target of miR-27b, significantly suppressed by miR-27b thereby downregulating the activities of MAPK/Erk signaling pathways in breast cancers. Taken together, the results strongly sustained that miR-27b would be a promising master role to overcome multiple chemoresistance in breast cancers through regulation both of PI3K/Akt signaling pathway and MAPK/Erk signaling pathway.

A genetic regulatory network is depicted in Fig. 7e, we had identified that downregulation of miR-27b, by hypermethylation in promoter region, is correlated with cell proliferation, evading apoptosis, and drug resistance. Mechanistic analysis indicated that miR-27b enhanced responses to PTX by directly targeting CBLB and GRB2 to inactivate both PI3K/Akt and MAPK/Erk signaling pathways. Further, miR-27b was identified as a molecular biomarker correlated with malignancy, poor survival and resistance to chemotherapeutic drugs of breast cancers. In conclusion, we provided a first insight into an important function of restoring miR-27b levels in breast cancers, and new therapeutic strategy about combinational use of miR-27b and anticancer drugs that would overcome the acquired multi-chemoresistances.

## Materials and methods

### Cell transfection and cell viability assay

The mature miR-27b mimics (miR-27b), miR-27b inhibitors and miR-NC (purchased from Ribobio, Guanghzou, China) were transfected into cells using LipofectamineTM 3000 according to the manufacturer’s instruction. For cell viability assay, cells were harvested, re-suspended to a final concentration of 1 × 10^4^ cells/ml, and evenly distributed into 96-well plates. After 12 h of incubation, the designated columns were treated with drug regimes for 72 h. Cell viability assays were performed by MTT assays. “Relative cell viability” = the viability of cells in drug-containing medium / the viability of cells in drug-free medium. “Relative cell viability” was further fitted to a dose-response curve to estimate the IC_50_ by the Graphpad Prism 6 software.

### *In vivo* studies

All animal procedures were performed according to protocols approved by the Animal Care Committee of Zhejiang University, school of medicine. For BCap37-xenograft experiment, BCap37 cells transfected with miR-27b inhibitors or NC inhibitors, and 3 × 10^6^ cells in 0.2 ml PBS were injected subcutaneously into the right flank regions of nude athymic mice (female, 5–6-weeks old, five mice, respectively). About 10 days later, 30 μg of lipofectamine3000-encapsulated miR-27b/NC inhibitors were injected intratumorally every 4 days for seven cycles. For Bads-200-xenograft experiment, the lentivirus vectors of miR-27b and blank vectors, purchased (Hanheng, Shanghai, China), were efficiently delivered into Bads-200 cells to establish stable overexpressing miR-27b cell lines and NC cell lines. A total of 5 × 10^6^ cells in 0.2 ml PBS were injected subcutaneously into the right flank regions of nude athymic mice (female, 5–6-weeks old, 10 mice, respectively). About 6 days later, each group of 10 mice were randomly divided into two subgroups and treated with PBS or PTX (10 mg/kg) through intraperitoneal injection every 6 days for total of five cycles. Both the maximum (*L*) and minimum (*W*) length of the tumor were measured using a slide caliper, and the tumor volume was calculated using the formula *V* = 1/2(*L* × *W*^2^). The curve of tumor growth was drawn based on tumor volume and corresponding time (days) after treatment. When the animals were terminated, the tumor tissues were removed and weighted.

### Dual-luciferase reporter assay

The 3′-UTRs of CBLB/GRB2 in Fig. 5a containing miR-27b putative target sites were amplified and cloned into psiCHECK-2 (Promega). A Fast Mutagenesis kit (VazymeBioTech) was used to mutate the miR-27b-binding sites of the CBLB and GRB2 3′-UTR vectors in Fig. 5a according to the manufacturer’s instructions. Dual-luciferase assays were performed using 1 × 10^4^ Bads-200 cells per well in a 96-well plate. Following attachment for 8 h, the cells were co-transfected with 50 ng respective reporter constructs with either miR-27b or NC (50 nM). After 48 h, the Reporter Assay System Kit (Promega, 017319) was used to measure the luciferase activity. Each transfectant was assayed in triplicates. Firefly luciferase activity was normalized to constitutiverenilla luciferase activity.

## Electronic supplementary material

Supplymentary figure 1

Supplymentary figure 2

Supplementary Figure Legends

Supplementary materials and methods

Supplementary Table
