# Supplementary figures and images for: miR-27b-3p inhibits proliferation and potentially reverses multi-chemoresistance by targeting CBLB/GRB2 in breast cancer cells
Source: Cell Death Dis. 2018 Feb 7;9(2):188. doi: 10.1038/s41419-017-0211-4 (PMC5833695; doi:10.1038/s41419-017-0211-4)

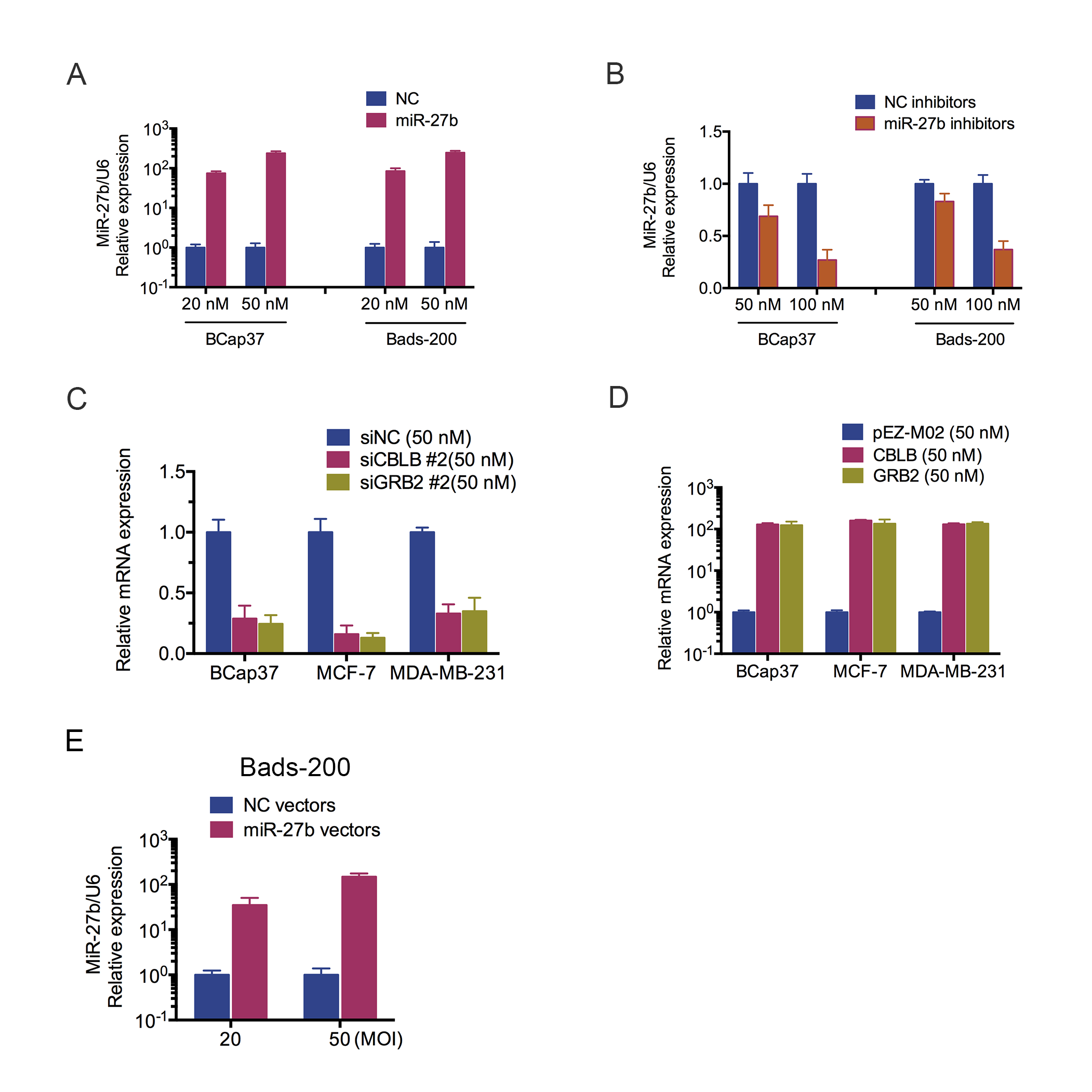

Supplement: Supplementary file 1 — Supplymentary figure 1 [file 41419_2017_211_MOESM1_ESM.tif]

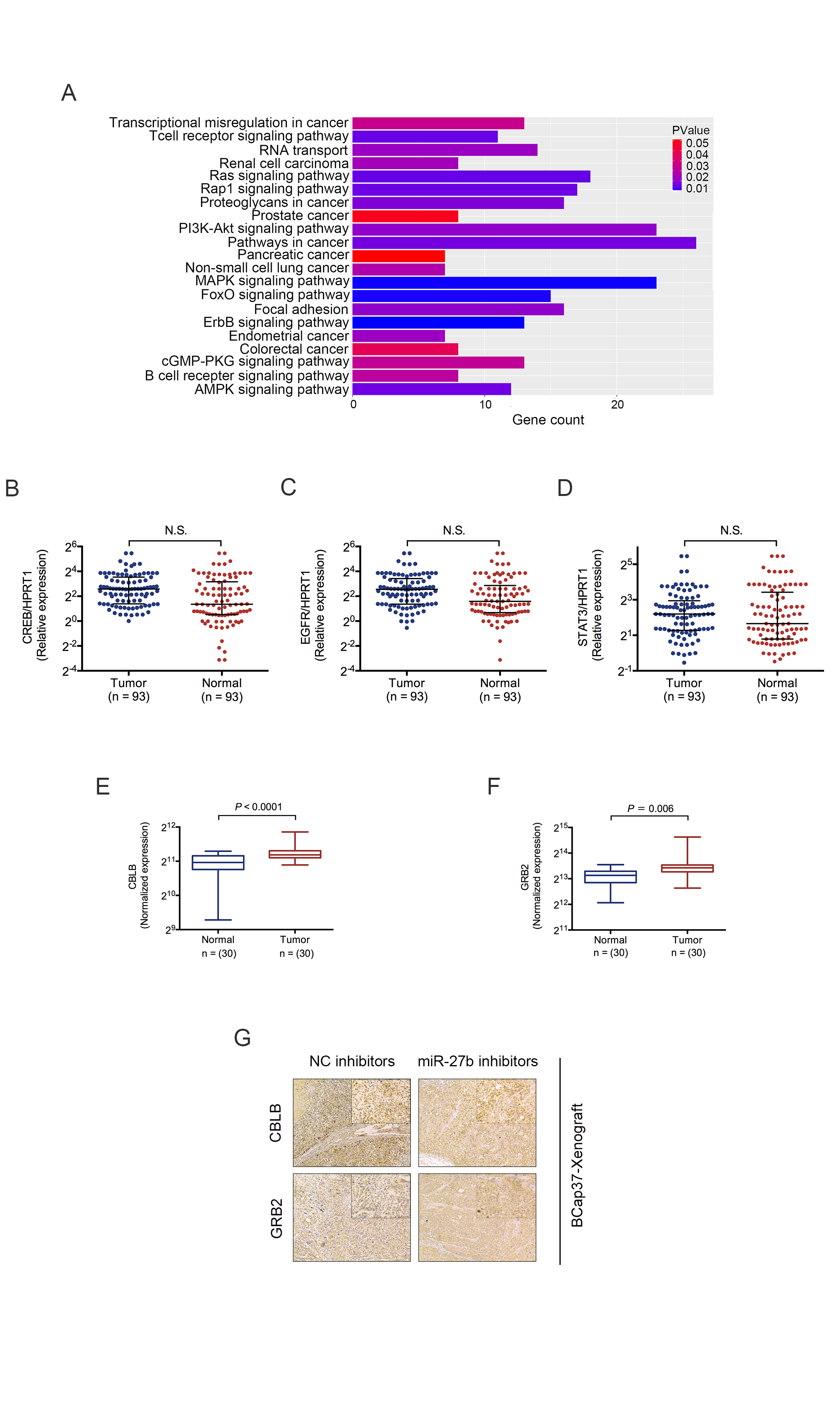

Supplement: Supplementary file 2 — Supplymentary figure 2 [file 41419_2017_211_MOESM2_ESM.tif]
