# Supplementary material for: miR-27b-3p inhibits proliferation and potentially reverses multi-chemoresistance by targeting CBLB/GRB2 in breast cancer cells
Source: Cell Death Dis. 2018 Feb 7;9(2):188. doi: 10.1038/s41419-017-0211-4 (PMC5833695; doi:10.1038/s41419-017-0211-4)
Supplement: Supplementary file 3 — Supplementary Figure Legends [file 41419_2017_211_MOESM3_ESM.doc]

**Supplementary Figure Legends**

Supplementary Figure 1.

(A) Quantitative PCR analysis. BCap37 and Bads-200 cell were transfected with miR-27b mimics (miR-27b) or NC mimics (NC) (20 or 50 nM). The expression levels of miR-27b were measured. Data was shown as mean ± SD. All assays were performed in triplicate and values represent the mean of three independent experiments.

(B) Quantitative PCR analysis. BCap37 and Bads-200 cells were transfected with miR-27b inhibitors or NC inhibitors (50 or 100 nM). The expression levels of miR-27b were measured.

(C) Quantitative PCR analysis. BCap37, MCF-7 and MDA-MB-231 cells were transfected with CBLB/GRB2 siRNA or siNC (50 nM). The expression levels of CBLB/GRB2 were measured.

(D) Quantitative PCR analysis. Bads-200, MCF-7 and MDA-MB-231 cells were transfected with CBLB/GRB2 overexpression plasmids or negative plasmids (50 nM). The expression levels of CBLB/GRB2 were measured.

(E) Quantitative PCR analysis. Bads-200 cells were transfected with miR-27b overexpression vectors (miR-27b) or blank vectors (NC) (MOI = 20, MOI = 50, respectively). The expression levels of miR-27b were measured.

Supplementary Figure 2.

(A) KEGG analysis was performed to invest functional pathways of potential target genes.

(B, C, D) Quantitative PCR analysis. The mRNA expression levels of CREB, EGFR and STAT3 in tumor tissues were compared with that in normal tissues in breast cancer patients, respectively (n = 93).

(E, F) Normalized expressions of CBLB/GRB2 were evaluated and compared in tumor tissues and matched normal tissues in luminal A-breast cancer patients in TCGA (n = 32).

(G) Representative images of tumor samples in BCap37-xenograft that were stained with CBLB and GRB2 by IHC. Scale bars: (main) 100 μm; (insets) 25 μm.
