# Supplementary material for: miR-27b-3p inhibits proliferation and potentially reverses multi-chemoresistance by targeting CBLB/GRB2 in breast cancer cells
Source: Cell Death Dis. 2018 Feb 7;9(2):188. doi: 10.1038/s41419-017-0211-4 (PMC5833695; doi:10.1038/s41419-017-0211-4)
Supplement: Supplementary file 4 — Supplementary materials and methods [file 41419_2017_211_MOESM4_ESM.doc]

**Supplementary materials and methods:**

Breast cancer cell lines

The human breast cell lines (HBL-100, Hs 578Bst, Hs 578T, BCap37, MDA-MB-231, SK-BR-3 and MCF-7) were purchased from the cell bank of the Chinese Scientific Academy. PTX-resistant Bads-200 cells were selected by continuous treatment of BCap37 cells for high concentrations of PTX in “time-increasing method”^.1^ HBL-100 cells were cultured in DMEM with 10% fetal bovine serum (FBS; Biological Industries, 04-0101-1, Cromwell, CT, USA). Hs 578Bst cells were cultured in DMEM with 50 ng/ml epidermal growth factor (EGF, Gibco, PHG0311) and 15% FBS. Hs 578T cells were cultured in Dulbecco's modified Eagle's medium (DMEM; Gibco, 12430047) supplemented with 0.01 mg/ml bovine insulin (Solarbio, I8040, Beijing, China) and 10% FBS. BCap37, MCF-7 and Bads-200 cells were cultured in Roswell Park Memorial Institute (RPMI) 1640 medium (Gibco, [31800105](https://www.thermofisher.com/order/catalog/product/31800105), Life Technologies, Carlsbad) with 10% FBS. MDA-MB-231 cells were cultured in Leibovitz’s L-15 medium (Gibco, 11415114) with 10% FBS. SK-BR-3 cells were cultured in McCoy's 5A medium (modified, Gibco, 16600082) with 10% FBS. The cell culture medium was changed every 2-3 days, and the cells were passaged with 0.25% trypsin-EDTA (Gibco, 25200056) and grown to 90% confluence. The cultures were kept at 37°C with 5% CO2 in a water-jacketed incubator (Thermo Scientific, Waltham, MA, USA). To maintain authenticity of the cell lines, frozen stocks were prepared from initial stocks, and every three months a new frozen stock was used for the experiments.

Breast cancer patients and tissue samples

The paraffin-embedded of primary breast cancer samples and paired adjacent normal tissues were obtained from patients who had undergone surgery at First Affiliated Hospital of Zhejiang University school of medicine (n = 93). In addition, the paraffin-embedded of primary breast cancer samples of patients in PTX-resistant groups and in PTX-sensitive groups were also obtained at First Affiliated Hospital of Zhejiang University school of medicine (n = 80). Clinical and pathological data were retrieved from clinical databases and from the original pathology reports, including basic information, survival time and tumor stage. This study was approved by the Ethical Committee of First Affiliated Hospital of Zhejiang University school of medicine, and informed consent was obtained from each patient prior to surgery.

Quantitative real-time PCR

Total RNA was extracted from tissues and cell lines using RNAiso plus Reagent (TaKaRa biotechnology,9109, Kusatsu, Japan). Total RNA was reverse transcribed into complementary DNA by using the PrimeScript RT Reageent kit (TaKaRa biotechnology, RR0037A). Quantitative-PCR (q-PCR) was performed in a Roche LightCycler480 II Real-Time PCR Detection System by using SYBR Premix Ex Taq (TaKaRa biotechnology, RR420A). Quantification of miRNAs was performed with a stem-loop real-time PCR. All PCR reactions were run in triplicate, miR-27b expression relative to U6, and gene expression relative to HPRT1, were calculated using the comparative threshold method (2-ΔΔCt).

Analysis of apoptosis

For apoptosis analysis, Bads-200 cells were plated in 6-well plates (4 × 10^5^ cells per well). 12 h later, Bads-200 cells were transfected with miR-27b and NC, 12 h after transfection, each groups were treated with PBS and different concentrations of PTX (1 μM; 1.5 μM) for 72 h. Similarly, BCap37 cells were plated in 6-well plates (2 × 10^5^ cells per well). 12 h later, BCap37 cells were transfected with miR-27b inhibitors and NC inhibitors, 12 h later, each groups were treated with PBS and different concentrations of PTX (4 nM; 8 nM) for 72 h. Moreover, BCap37 cells were co-transfected with miR-27b inhibitors and CBLB/GRB2 siRNA (miR-27b inhibitors 100 nM; siCBLB or siGRB2 50 nM). 12 h after transfection the cell culture medium was changed and cells were cultured for another 2 days. AnnexinV/PI apoptosis detection kit (Beyotime, Haimen, China) was used to detect cell apoptosis according to the manufacturer’s instructions, and the percentage of apoptotic cells was determined by flow cytometryanalysis which was performed in a BD FACSCalibur™ flow cytometry system.

Immunohistochemistry staining

Antibodies specific against CBLB (abcam 1:500), GRB2 (abcam 1:500) and Ki67(abcam 1:5000) were used for the IHC of 4-μm-thick paraffin embedded sections of xenograft tumor samples. The staining was visualized using the DAKO Envision kit according to the instructions of the manufacturer (DAKO, CA). Slides were photographed using an optical microscope (Olympus).

Western blotting

Total proteins were extracted from cell lines, and the protein concentration was determined using a BCA protein assay kit (Beyotime Biotec, China). Proteins samples were fractionated using 6-10% SDS-PAGE gels and transferred to PVDF membranes (Millipore, NY, USA)，and blocked for 1 hour with 5% non-fat milk and were then incubated at room temperature with primary antibodies: rabbit anti-human CBLB (1:500; Abcam, UK), rabbit anti-human GRB2, AKT, p-AKT, ERK, p-ERK, Bcl-xl and Bcl-2 (1:2000; Abcam, UK). mouse anti-human GAPDH antibodies (1:2000, Abcam) were used as endogenous controls. Protein expression levels were detected with ECL detection solution (Apexbio, Houston, USA) by G-BOX System with GeneSnap software (Syngene). Experiments were repeated independently at least three times.

The Cancer Genome Atlas (TCGA) database

For patient analysis, all data were retrieved from TCGA data portal (<https://genome-cancer.ucsc.edu/)> updated by the end of March 31, 2017. All miRNA expression, CBLB and GRB2 mRNA expression, DNA methylation data were used in level three. For breast cancers, there are 32 paired samples both in tumor tissues and adjacent normal tissues with miR-27b expression, while in which 18 paired samples contain DNA methylation data. Early-stage breast cancer patients (TNM stage I and II) with miR-27b expressions and survival informations were obtained to describe the correlation between miR-27b expression and survival (n = 262). In figure 1H, data on 350 patients were obtained to describe the correlation between miR-27b expression and breast cancer maliganancy.

According to the information provided by TCGA, miRNA expressions were measured using IIIumina GASeq/HiSeq 2000 Sequencing. The miRNA expression levels were quantified by reads per million miRNA mapped. DNA methylation was measured using IIIumina infinium Human DNA Methylation 450. DNA methylation level at each CpG site on the miR-27b gene promoter was calculated by β value (β = methylated raw value / (methylated raw value raw value +100)), raging from 0, in the case of completely unmethylated sites, to 1, in completely unmethylated sites. The median β value of CpG sites on the miR-27b gene promoter was used to assess the methyloation status of each human sample.

Statistical analysis

All experiments were performed in triplicate and the data are expressed as mean ± standard deviation (SD). Statistical analyses were performed with the GraphPad Prism Software (GraphPad). A two-tailed Student’s t-test was used to evaluate the differences between two groups of data and one-way ANOVA followed by Tukey’s posttest was used to compare the means of three independent groups. The Kaplane-Meier method and log-rank test were used to evaluate the correlation between miR-27b expression and patient survival. The correlation between miR-27b expression and clinicopathological characteristics was analyzed by Pearson correlation analysis.

*, P < 0.05; **, P < 0.01; *** P < 0.001. P values < 0.05 were considered statistically significant.

**References:**

1.Jeong JY, Kang H, Kim TH, Kim G, Heo JH, Kwon AY, et al. MicroRNA-136 inhibits cancer stem cell activity and enhances the anti-tumor effect of paclitaxel against chemoresistant ovarian cancer cells by targeting Notch3. CANCER LETT. [Journal Article]. 2017 2017-02-01;386:168-78.
