# Supplementary material for: miR-27b-3p inhibits proliferation and potentially reverses multi-chemoresistance by targeting CBLB/GRB2 in breast cancer cells
Source: Cell Death Dis. 2018 Feb 7;9(2):188. doi: 10.1038/s41419-017-0211-4 (PMC5833695; doi:10.1038/s41419-017-0211-4)
Supplement: Supplementary file 5 — Supplementary Table [file 41419_2017_211_MOESM5_ESM.docx]

Table S1 Sequences for primers used in this study

| **Name** | **Sequence** |
| --- | --- |
| **q-PCR primers**  CBLB-F  CBLB-R  GRB2-F  GRB2-R  CREB-F  CREB-R  EGFR-F  EGFR-R  STAT3-F  STAT3-R  MAPK1-F  MAPK1-R  KRAS-F  KRAS-R  NF1-F  NF1-R  TGFBR1-F  TGFBR1-R  FOXO3-F  FOXO3-R  GSK3B-F  GSK3B -R  VAV3-F  VAV3-R  VEGFC-F  VEGFC-R  **Primers for 3’UTR**  CBLB-F  CBLB-R  GRB2-F  GRB2-R  mut-CBLB-F  mut-CBLB-R  mut-GRB2-F  mut-GRB2-R | CAACGGGTTCACCAAAGTCT  GGATTGGTGGAGGTCTTTCA  TTCCTCTGGGTGGTGAAGTT  CTGCTGTGGCACCTGTTCTA  AGCGGAGTGTTGGTGAGTGA  TGGTACAAGCTCCTCCGTCA  AATGCGTGGACAAGTGCAAC  TGTGCAGGTGATGTTCATGG  CATCCTGGCTAACACGGTGA  AAGCGATTCTCCTGCCTCAG  CCGCTCGAGAGTATGTACTTCAGTGCA  ATAAGAATGCGGCCGCATAGCACATACCT  CAGGCTGGTCTCGAACTCCT  CGGTGACTGGCATCTGGTAG  TGCCTTCCGTTCCAGTTACC  CATGCCTCCATGATCTCCAA  GGTTCCGTGAGGCAGAGATT  CCAACCAGAGCTGAGTCCAA  CAGCCTGACCAACATGGAGA  AAGCGATTCTCCTGCCTCAG  TTCCTCCTCATGCTCGGATT  CAGGTGGAGTTGGAAGCTGA  GCCATCGCTCGGTATGACTT  TCTCCTCTCCACCAGCCATT  AATCACACTTCCTGCCGATG  CTTGTTCGCTGCCTGACACT  CCGCTCGAGGAAGAGGCTTGGGAGTGC  AAATATGCGGCCGCTGTGGACAGCAAGAGAGT  aattctaggcgatcgctcgagTTCCTCATCCTGCTTCTCTTTTCC  attttattgcggccagcggccgcTGAAGAATTCATTGTGTATTTATTATTCACA  CgacactAATGCTTTACCTTGTTTACAGTTTGG  GGTAAAGCATTagtgtcGTTCAAAGTTCAAGGGAAGTAAACG  GTAGTGATTAtgacacttTAATAAATACACAATGAATTCTTCA  aagtgtcaTAATCACTACCTACCAAATGCTATCCG |

Table S2. Sequences for siRNAs used in this study

| Name | Sequence |
| --- | --- |
| **siRNAs**  siCBLB# 1(sense)  siCBLB# 1(antisense)  siCBLB# 2(sense)  siCBLB#2 (antisense)  siCBLB# 3(sense)  siCBLB#3 (antisense)  siGRB2#1 (sense)  siGRB2 # 1(antisense)  siGRB2#2 (sense)  siGRB2 #2 (antisense)  siGRB2#2 (sense)  siGRB2 #2 (antisense) | CGGGCAAUAAGACUCUUUAUU  UAAAGAGUCUUAUUGCCCGUU  GGGCUUUAGAAUUUAGAAUUU  AUUCUAAAUUCUAAAGCCCUU  CCCAGGGCUUUAGAAUUUAUU  UAAAUUCUAAAGCCCUGGGUU  GGCCCUCUUUGACUUUGAUUU  AUCAAAGUCAAAGAGGGCCUU  GGGCUGGUAUUCUCUCUAUUU  AUAGAGAGAAUACCAGCCCUU  CUCCUACUCUGUGUAAAUAUU  UAUUUACACAGAGUAGGAGUU |

Table S3. The sensitivity of Bcap37 and Bads-200 cells to anticancer drugs

| Drug | BCap37  IC50 (nM) | Bads-200  IC50 (nM) |
| --- | --- | --- |
| Paclitaxel | 4.62 ± 0.86 | 12500 ± 1137.5 |
| Cisplatin | 1230 ± 160 | 1450 ± 210 |
| Doxorubicin | 102.4 ± 8.2 | 10500 ± 650 |
| Gemcitabine | 536.6 ± 60.2 | 9980 ± 430 |
| Fluoruouracil | 13750 ± 6200 | 22800 ± 1750 |
